# Vibrational Raman and IR data on brown hair subjected to bleaching

**DOI:** 10.1016/j.dib.2021.107439

**Published:** 2021-09-30

**Authors:** Michele Di Foggia, Carla Boga, Gabriele Micheletti, Benedetta Nocentini, Paola Taddei

**Affiliations:** a Department of Biomedical and Neuromotor Sciences, Alma Mater Studiorum – Università di Bologna, Via Belmeloro 8/2, Bologna 40126, Italy; b Department of Industrial Chemistry ‘Toso Montanari’, Alma Mater Studiorum – Università di Bologna, Viale Del Risorgimento 4, Bologna 40136, Italy

**Keywords:** Hair keratin, Bleaching, Raman spectroscopy, IR spectroscopy, Secondary structure, Cysteic acid, Disulfide bridges

## Abstract

Brown human hair was bleached three times (45 min × 3) and four times (45 min × 3 + 15 min) with commercial formulations containing persulfate salts and hydrogen peroxide. The hair samples were characterized by Raman and IR spectroscopy in the Attenuated Total Reflectance (ATR) mode to gain more insights into the possible secondary structure and C_α_-C_β_-S-S-C_β_-C_α_ conformational changes induced by bleaching. The latter were evaluated through band-fitting procedures; the relative content of the disulfide bridges and oxidized sulfur species (cysteic acid, Bunte salt, cystine oxides) was assessed. The observed conformational changes could be significant in developing restoring agents to be used after hair decoloration. The use of two different spectroscopic techniques allowed to discriminate the information coming from the cortical region of hair (Raman) and the cuticle (ATR/IR).

This article refers to “Structural investigation on damaged hair keratin treated with α,β-unsaturated Michael acceptors used as repairing agents” (Di Foggia et al., Int. J. Biol. Macromol. 167 (2021) 620–632 https://doi.org/10.1016/j.ijbiomac.2020.11.194).

**Specifications Table**

**Table:** 

Subject	Chemistry
Specific subject area	Analytical Chemistry: Spectroscopy
Type of data	Tables, Graphs, Figures
How data were acquired	Fourier-Transform FT-Raman spectroscopy (Bruker MultiRam FT-Raman spectrometer); FT-IR spectroscopy (Bruker Alpha FT-IR spectrometer) in Attenuated Total Reflectance (ATR) mode (Platinum ATR single reflection diamond module). Curve-fitting analysis (OPUS version 6.5 program); Statistical analysis (R statistical software version 3.5.3; GNU GPL license).
Data format	Raw and analyzed
Parameters for data collection	Brown human hair was bleached three times (45 min × 3) and four times (45 min × 3 + 15 min) with commercial formulations (1:1 mixture of Lunex Ultra Cream and Uni.Color Oxi, Kemøn S.p.A., Perugia, Italy), then washed with Actyva Colore Brillante Shampoo, rinsed with water and dried with a hairdryer.
Description of data collection	Raman and IR spectra (in the ATR mode) were recorded on the hair samples bleached three times and four times. The IR spectrum of brown hair was recorded for comparison. The high concentration of melanins in brown hair made impossible the registration of the Raman spectrum of this sample.
Data source location	Bologna, Italy, University of Bologna (44.49381, 11.33875).
Data accessibility	With the article
Related research article	M. Di Foggia, C. Boga, G. Micheletti, B. Nocentini, P. Taddei, Structural investigation on damaged hair keratin treated with α,β-unsaturated Michael acceptors used as repairing agents, Int. J. Biol. Macromol. 167 (2021) 620–632 10.1016/j.ijbiomac.2020.11.194

**Value of the Data**

• ATR/IR and Raman spectroscopic data evidence the conformational changes and the degradation of hair fibers after multiple bleaching treatments
• The spectroscopic data could be significant for academic and industrial researchers in the development of hair and wool restoring agents
• The presented data could be a starting point for a deeper investigation of the effects of hair treatments.

## 1 Data Description

**Fig. 1:**
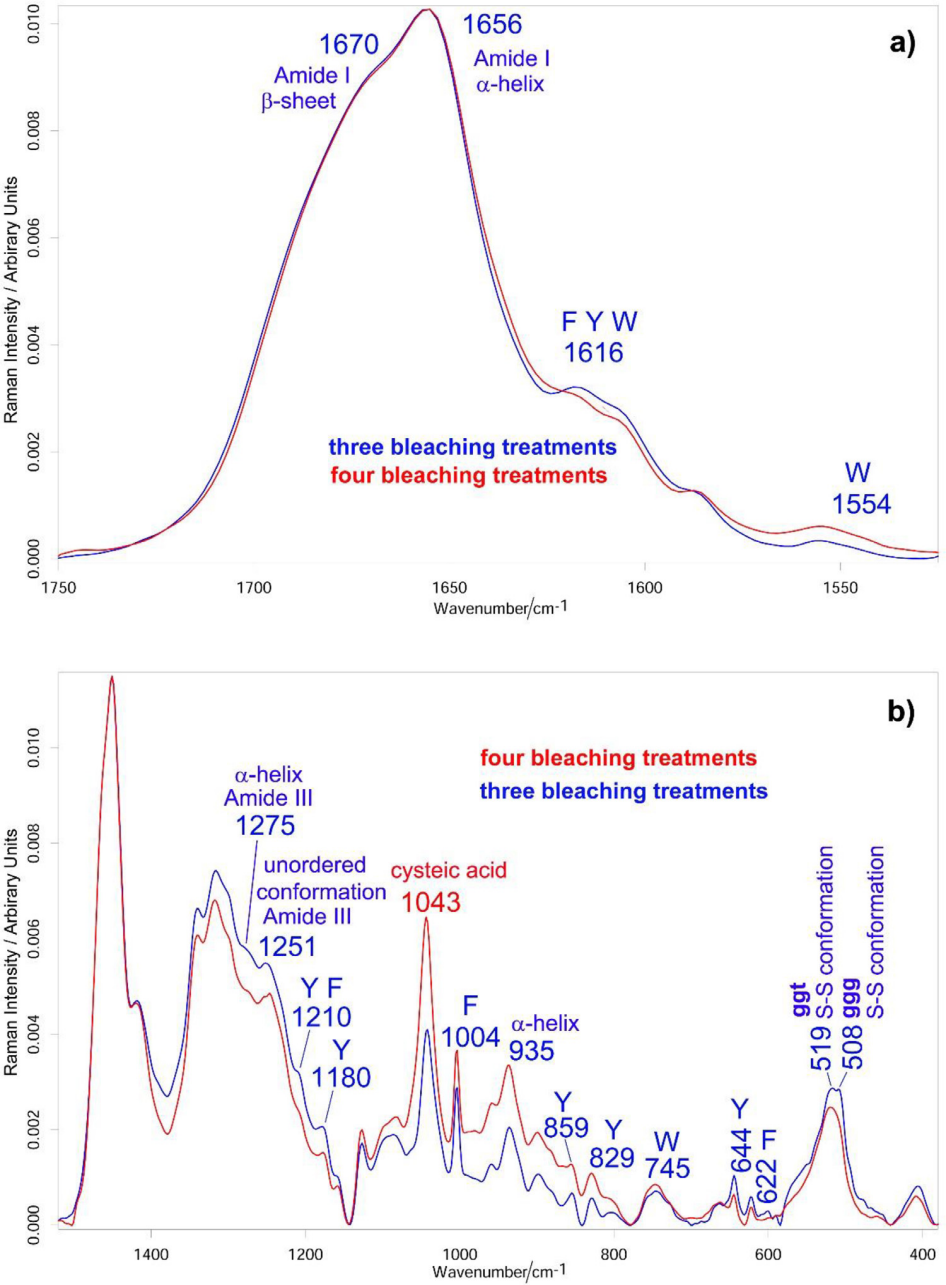
Average Raman spectra of the hair locks that underwent three and four bleaching treatments: (a) 1750–1520 cm^−1^ range; (b) 1500–380 cm^−1^ range. The bands assignable to specific amino acids are indicated: *F* = phenylalanine; *Y* = tyrosine; *W* = tryptophan; ggg and ggt = *gauche-gauche-gauche* and *gauche-gauche-trans* conformations of the C_α_-C_β_-S-S-C_β_-C_α_ system. The spectra reported in the figures are normalized to the intensity of the band at 1450 cm^−1^ (CH_2_ bending), which was chosen as an internal standard [4,5].

Fig. 1 shows the average Raman spectra of the hair locks that underwent three and four bleaching treatments (see Supplementary Material, “Fig. 1” folder, for raw data). Assignments have been given according to the literature [1], [2], [3], [4], [5], [6].

**Fig. 2:**
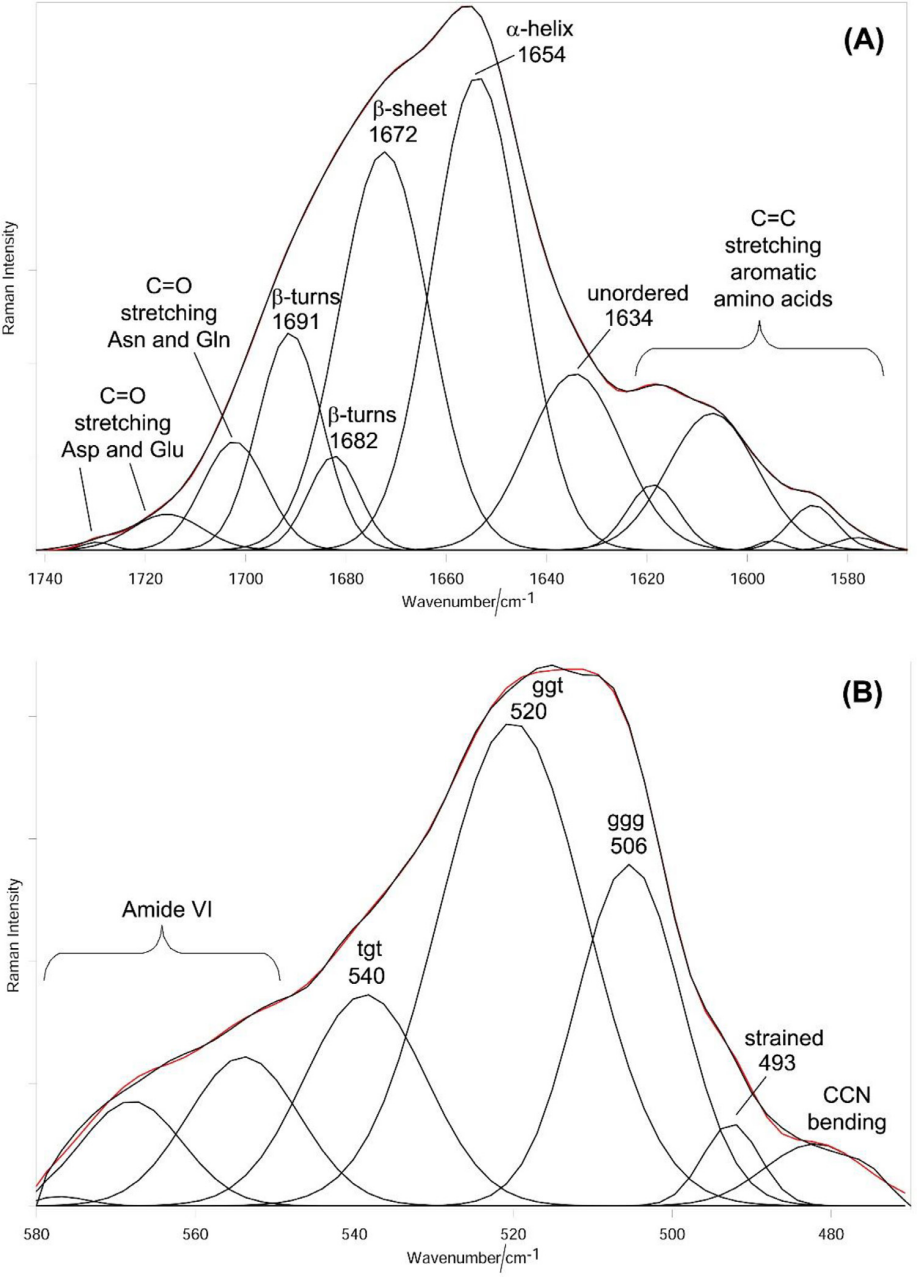
Raman spectrum in the Amide I (A) and S-S stretching (B) ranges of the hair lock bleached three times fitted into its components. The black and red curves represent the experimental and the fitted spectra, respectively. The curve-fitting procedure was carried out by using as starting positions the frequencies of the maxima of the fourth-derivative spectra (obtained with 13-point smoothing). Bands wavenumbers and assignments [4], [5], [6] are indicated (ggg, ggt and tgt = *gauche-gauche-gauche, gauche-gauche-trans* and *trans-gauche-trans* conformations of the C_α_-C_β_-S-S-C_β_-C_α_ system).

**Fig. 3:**
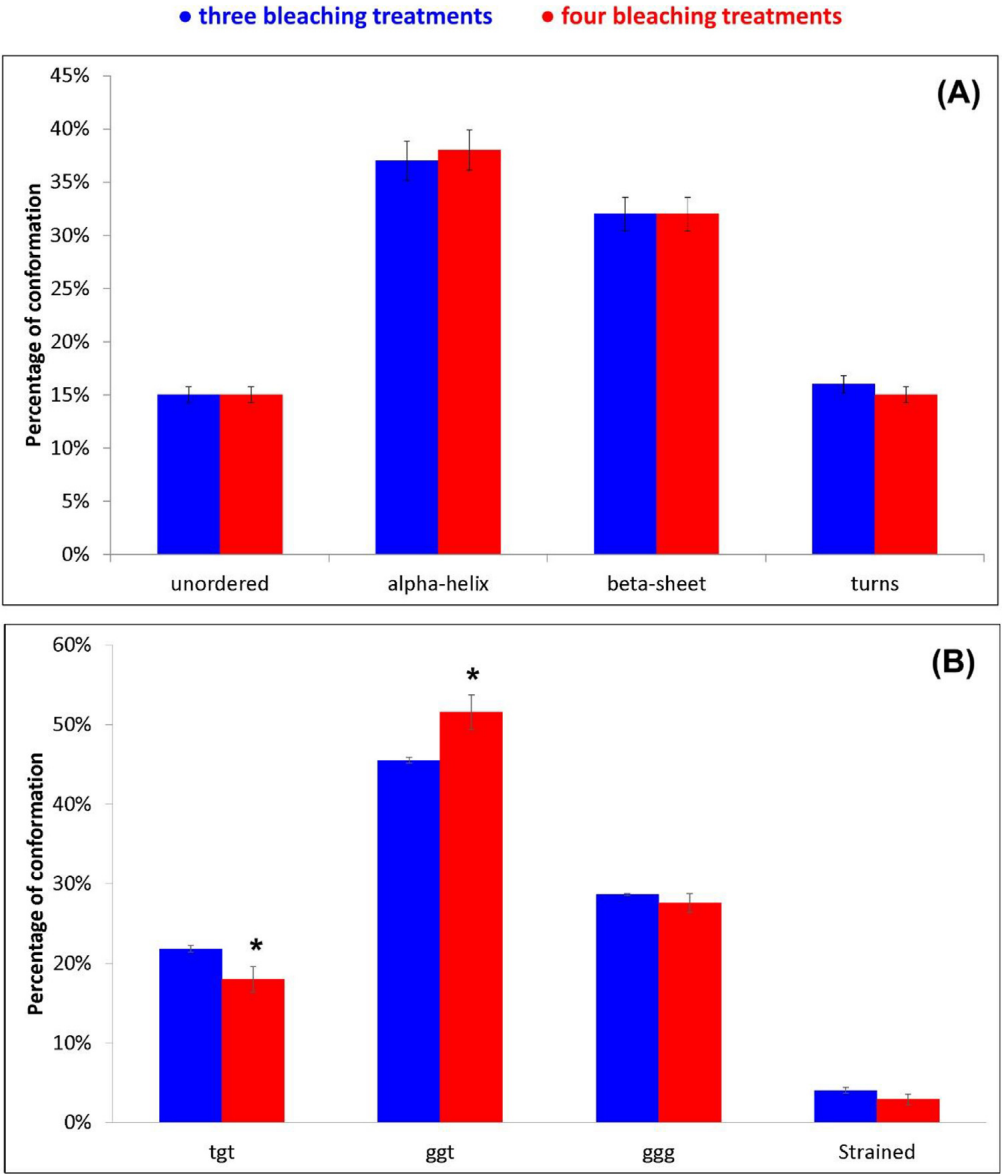
(A) Percentages (average ± standard deviation) of secondary structure conformations as obtained by the curve fitting of the Raman Amide I range of the hair locks that underwent three and four bleaching treatments. (B) Percentages (average ± standard deviation) of strained, *gauche-gauche-gauche* (ggg), *gauche-gauche-trans* (ggt), and *trans-gauche-trans* (tgt) C_α_-C_β_-S-S-C_β_-C_α_ conformations as obtained by the curve fitting of the Raman S-S stretching range of the same samples. Asterisks indicate statistically significant differences between the two treatments.

Only slight changes in the relative intensity of the Amide bands were observed upon the fourth decoloration, suggesting only minor conformational rearrangements, as confirmed by the Amide I fitting data (Figs. 2A and 3A, see Supplementary Material, “Fig. 2A” and “Fig. 3A” folders, for raw data). The main conformation in the cortical region of both the bleached samples (i.e. the main region of which the Raman spectra are representative), accounting for about 37-38%, was α-helix. As expected, the A_935_/A_1450_ ratio (identified as a maker of the content of this structure) remained constant at a value of 0.049 ± 0.004.

**Fig. 4:**
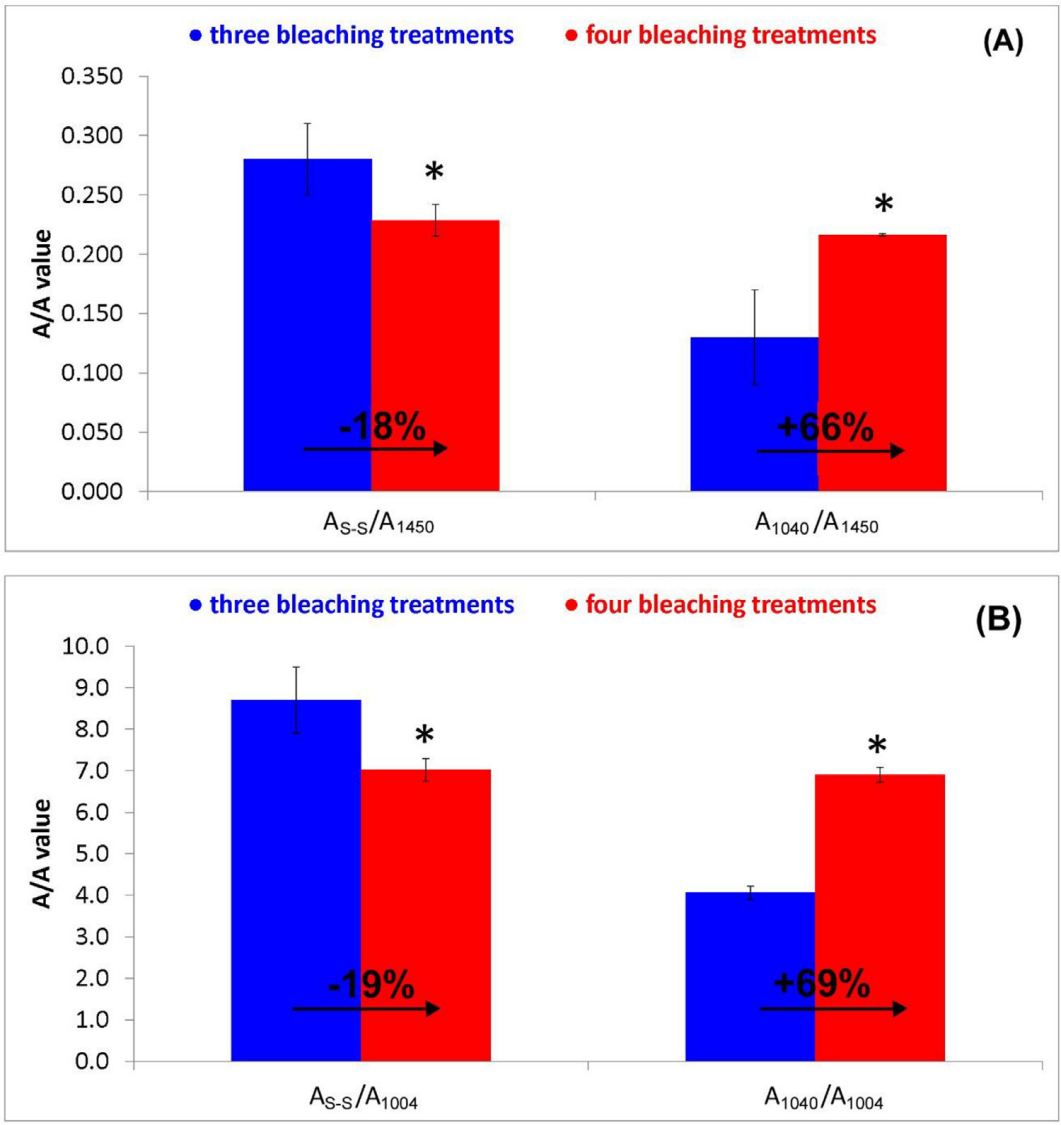
Trend of the Raman A_s-s_/A_1450_, A_1040_/A_1450_ (A) and A_s-s_/A_1004_, A_1040_/A_1004_ (B) ratios (average ± standard deviation) as calculated from the Raman spectra recorded on the hair locks that underwent three and four bleaching treatments. Percentage variations are reported. Asterisks indicate statistically significant differences between the two treatments.

The relative content of the disulfide bridges (broad band at about 500 cm^−1^, Fig. 1) and cysteic acid (band at about 1040 cm^−1^, Fig. 1) in the cortical region was evaluated through the Raman A_s-s_/A_1450_, A_1040_/A_1450_, A_s-s_/A_1004_, and A_1040_/A_1004_ ratios, and their trend is reported in Fig. 4 (see Supplementary Material, “Fig. 4” folder, for raw data). Going from the sample bleached three times to that bleached four times, the A_s-s_/A_1450_ and A_s-s_/A_1004_ ratios significantly decreased, whilst A_1040_/A_1450_ and A_1040_/A_1004_ noticeably increased. The further S-S cleavage induced by the fourth bleaching treatment and the formation of cysteic acid occurred with no parallel change in the α-helix content (Fig. 3A).

The percentage variations of the A_s-s_/A_1450_ and A_s-s_/A_1004_ ratios (Fig. 4) were in perfect agreement with each other, and the same result was found by comparing A_1040_/A_1450_ and A_1040_/A_1004_; therefore, the use of an internal standard band rather than the other is irrelevant. Moreover, it may also be deduced that the bleaching treatment for a fourth time did not alter the CH_2_ content in the cortical region with respect to the sample bleached three times.

The fitting data of the SS band at about 500 cm^−1^ were used to investigate the different conformations of the C_α_-C_β_-S-S-C_β_-C_α_ system (Figs. 2B and 3B, see Supplementary Material, “Fig. 2B” and “Fig. 3B” folders, for raw data). Upon three bleaching treatments, the main S-S conformation had already become the *gauche-gauche-trans* one instead of the *gauche-gauche-gauche* conformation, which is the main one in natural keratins (i.e., the thermodynamically most stable). Upon the fourth bleaching treatment, the *gauche-gauche-trans* content further increased at the expense of the *trans-gauche-trans* and (to a lesser extent) *gauche-gauche-gauche* conformations.

Some bands attributable to specific amino acid side-chains (Fig. 1) decreased in intensity upon the fourth bleaching. In particular, the tyrosine bands underwent this behavior, confirming the removal of this amino acid by the bleaching treatment [5].

**Fig. 5:**
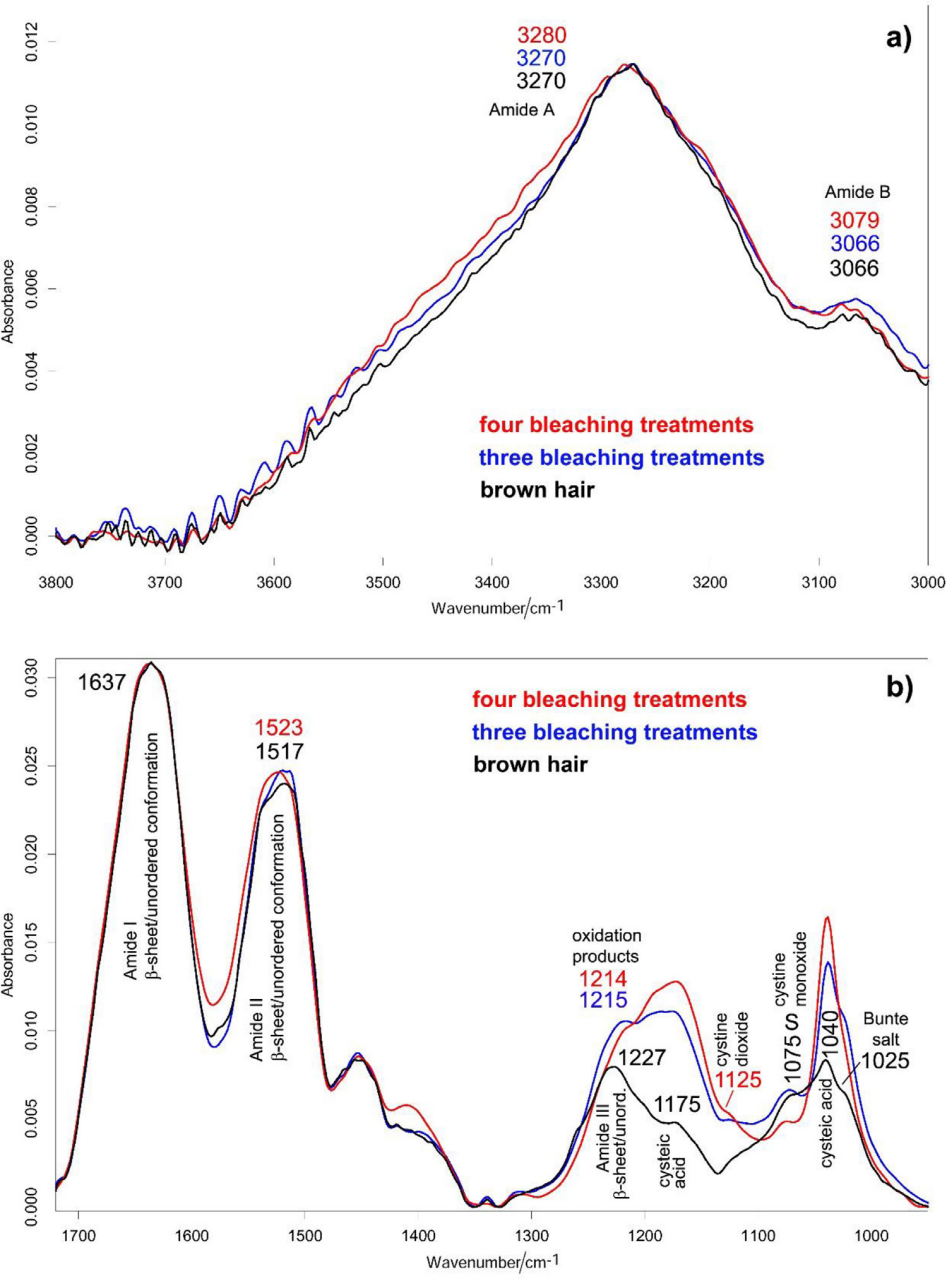
Average ATR-IR spectra of black hair, as well as after three and four bleaching treatments: (a) 3800–3000 cm^−1^ range; (b) 1720–950 cm^−1^ range. The main band assignable to serine (S) is indicated. The spectra reported in both figures are normalized to the absorbance of the Amide I band, except for the spectrum corresponding to four bleaching treatments in figure (a), which was rescaled to the same absorbance of Amide A as the others.

Fig. 5 shows the ATR-IR spectra of brown hair as well as after three and four bleaching treatments (see Supplementary Material, “Fig. 5” folder, for raw data). Assignments have been given according to the literature [4,[7], [8], [9], [10], [11], [12], [13]]. The contribution of OH stretching and bending modes of water to the Amide A and Amide I ranges, respectively, may be considered negligible since dried hair samples were analyzed.

The ATR spectrum is representative of the cuticle, whose main conformation appeared β-sheet/unordered [7], in agreement with other IR studies on keratins [4,8].

**Fig. 6:**
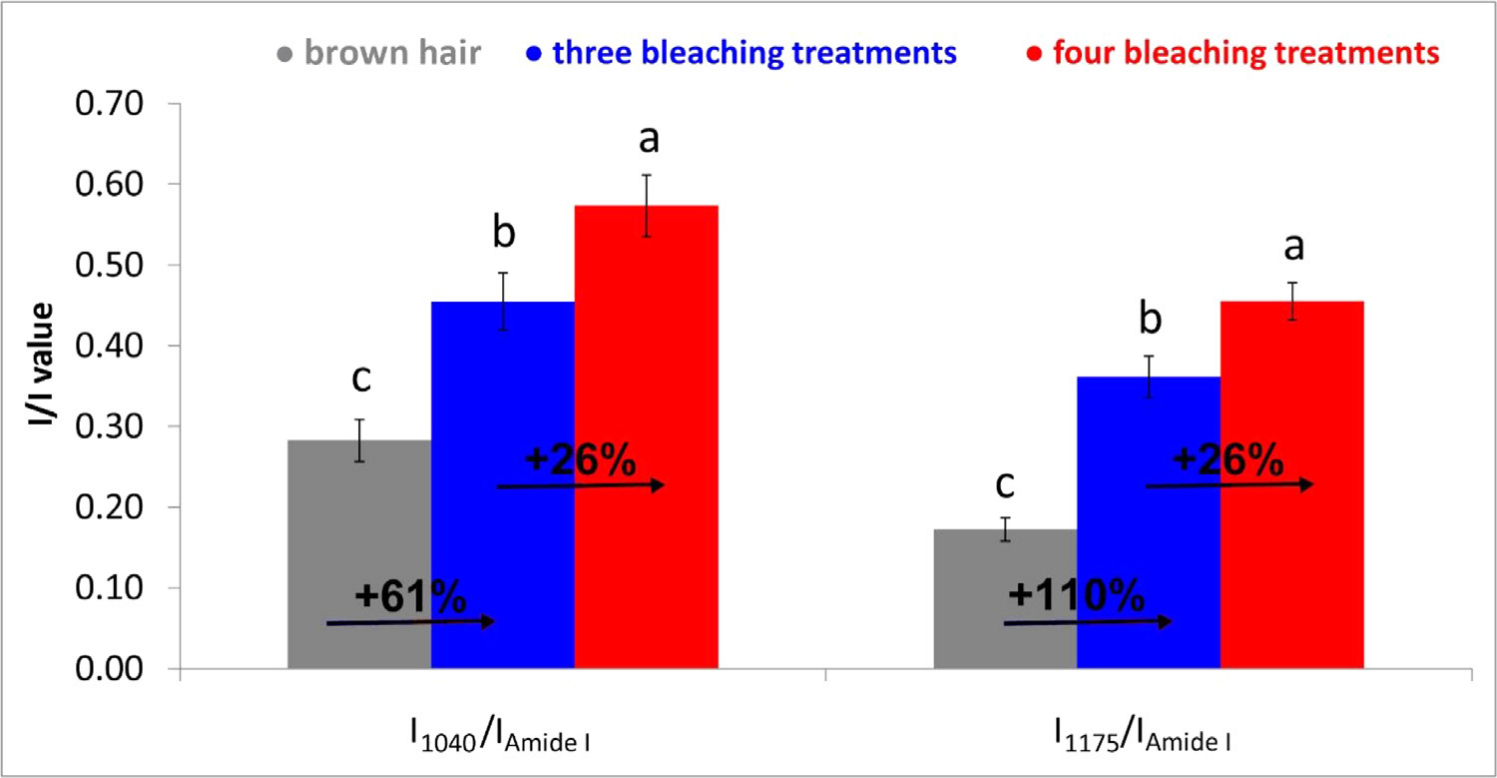
Trend of the IR I_1040_/I_Amide I_ and I_1175_/I_Amide I_ ratios (average ± standard deviation) as calculated from the IR spectra recorded on black hair, as well as after three and four bleaching treatments. Percentage variations are reported. For each ratio, different letters on histogram bars represent statistically significant differences.

Oxidation products, mainly cysteic acid (bands at about 1040 and 1175 cm^−1^) and secondarily Bunte salt (band at about 1025 cm^−1^) were also identified in the spectrum of starting brown hair due to weathering (exposure to sunlight, wind, grooming) [12]. Upon three and four bleaching treatments, these products further increased their contents. The I_1040_/I_Amide I_ and I_1175_/I_Amide I_ ratios were calculated to follow the relative content of cysteic acid upon the treatments (Fig. 6, see Supplementary Material, “Fig. 6” folder, for raw data). The most significant increase in this oxidation product was observed going from brown hair to the sample bleached three times. The percentage increase measured by IR spectroscopy going from three to four bleaching treatments (Fig. 6) was lower than the corresponding one obtained by Raman spectroscopy (Fig. 4). Evidently, at this stage (i.e., when the hair has undergone three bleaching treatments), the further bleaching should have affected the cortical region more than the cuticle, as also observed by SEM analyses [14].

Going from brown hair to that bleached three times, the Bunte salt content increased at the same rate as cysteic acid; actually, the A_1025_/A_1040_ absorbance ratio (calculated after curve-fitting) between their marker bands remained constant (Table 1, see Supplementary Material, “Table 1” folder, for raw data).

**Table 1:** A_1025_/A_1040_ ratios (average ± standard deviation) as obtained by the curve-fitting analysis of the IR spectra of the samples under study. Different letters represent statistically significant differences (*p* < 0.05).

Sample	A_1025_/A_1040_ (average ± standard deviation)
Brown hair	0.78 ± 0.11 a
Bleached three times	0.78 ± 0.18 a
Bleached four times	0.32 ± 0.05 b

IR spectroscopy also revealed other keratin oxidation products (intermediates), such as cystine monoxide and dioxide (Fig. 5).

Upon the fourth decoloration, the serine band at 1075 cm^−1^ decreased in intensity, confirming the involvement of this amino acid in the hair degradation process induced by bleaching with H_2_O_2_ [5]. Conformational changes towards a more disordered structure occurred, as revealed by the shifts of Amide II, Amide A, and B modes and the strengthening of Amide II if compared with Amide I. The Bunte salt band at 1025 cm^−1^ was detected as a shoulder, and the A_1025_/A_1040_ absorbance ratio significantly (*P*< 0.05) decreased with respect to the sample bleached only three times (Table 1), suggesting that upon the fourth bleaching, the cysteic acid content increased at a higher rate than the Bunte salt, and the distribution of the oxidation products changed.

## 2 Experimental Design, Materials and Methods

To gain information on the effect of bleaching on brown human hair, the treatment was first carried out three times to obtain a homogeneous decoloration grade, independently of the nuance of the starting material. This recalls the routine operation carried out in hairdresser saloons. Then, a further bleaching treatment was performed, and the fibers were analyzed through ATR/IR and Raman spectroscopy.

### 2.1 Materials

Lunex system Ultra Cream and Actyva Colore Brillante Shampoo were produced by Kemøn S.p.A. (Perugia, Italy). Their composition is reported in Table 2. Brown human hair was kindly provided by B. Nocentini.

**Table 2:** Composition of the commercial formulations used in the study.

	Commercial name	Composition
**Commercial formulations for bleaching procedure**	**LUNEX System Ultra Cream**	potassium persulfate, ammonium persulfate, sodium metasilicate, sodium silicate, sodium lauryl sulfoacetate, sodium persulfate, carbomer, ethylene/propylene/styrene copolymer, sodium stearate, octyldodecyl myristate, paraffinum liquidum (mineral oil/huile minerale), sodium chloride, sodium sulfate, dimethicone, bisabolol, hydrolyzed keratin, tetrasodium EDTA, silica, hydrated silica, hydroxyethylcellulose, butylene/ethylene/styrene copolymer, sorbitol, aqua (water/eau), Cl 77007 (ultramarines), CI 60730 (acid violet 43).

**UNI.COLOR OXI 40** (oxidant solution with a 40 vol content of H_2_O_2_)	aqua (water/eau), hydrogen peroxide, paraffinum liquidum (mineral oil/huile minerale), cetearyl alcohol, ceteareth-20, cetyl alcohol, oxyquinoline sulfate, etidronic acid.

**Commercial shampoo**	**Actyva Colore Brillante Shampoo**	aqua (water/eau), sodium C14-16 olefin sulfonate, sodium cocoamphoacetate, cocamide DEA, carbomer, cocamidopropyl betaine, dimethicone, dicaprylyl ether, lauryl alcohol, propylene glycol, polysilicon-19, dipropylene glycol, laureth-3, bitter cherry seed oil PEG-8 esters, Vaccinium myrtillus extract, glycol distearate, polyquaternium-10, guar hydroxypropyltrimonium chloride, triethanolamine, methylparaben, ethylparaben, propylparaben, imidazolidinyl urea, laureth-8, succinoglycan, glycerin, sodium methylparaben, sodium dehydroacetate, sorbic acid, tetrasodium EDTA, parfum, limonene, coumarin, alpha-isomethyl ionene, benzyl salicylate, hydroxycitronellal, citronellol, hydroxyisohexyl 3-cyclohexene carboxaldehyde, benzyl benzoate, Cl 45,100, Cl 42090

### 2.2 Preparation of the samples for vibrational analyses

The samples were prepared by following the steps described below.

1. A lock of brown human hair (10 g) was immersed for 45 min at 35 °C in a 1:1 mixture of Lunex Ultra Cream and Uni.Color Oxi, then washed with Actyva Colore Brillante Shampoo, rinsed with water, and dried with a hairdryer.
2. The procedure was repeated three consecutive times.
3. A part of the above hair lock bleached three times was subjected to further bleaching (*e.g.,* 0.35 g of hair were treated with 10 g of Lunex Ultra Cream and 10 g of UniColor Oxi 40 vol). The lock was wrapped in aluminum foil and placed in an oven at 35 °C for 15 min.
4. The lock was washed with Actyva Colore Brillante Shampoo, rinsed with water, and dried with a hairdryer.
5. Each human hair sample used for Raman and ATR/FT-IR analyses was a lock of about 12 cm in length, 0.3 cm in diameter, and 0.35 g in weight.
6. The dried locks subjected to three and four bleaching treatments were analyzed by Raman and ATR/FT-IR analyses, and the results were compared.

### 2.3 Vibrational spectra acquisition

Raman spectra were recorded using a Bruker MultiRam FT-Raman spectrometer equipped with a cooled Ge-diode detector. The excitation source was an Nd^3+^-YAG laser (1064 nm) in the backscattering (180°) configuration. The focused laser beam diameter was about 100 µm, the spectral resolution 4 cm^−1^, the laser power at the sample about 80 mW. The number of scans was 5000 for each spectrum.

It must be recalled that the high concentration of melanins in brown hair made impossible the registration of the Raman spectrum of this sample; actually, melanins are highly fluorescent and are responsible for worsening the Raman signal [13,14]. The bleaching treatment decomposed melanin granules [15], allowing the recording of good Raman spectra.

IR spectra were recorded on a Bruker Alpha Fourier Transform FTIR spectrometer, equipped with a Platinum Attenuated Total Reflectance (ATR) single reflection diamond module (penetration depth 2 µm) and a Deuterated Lanthanum α-Alanine doped TriGlycine Sulfate (DLaTGS) detector; the spectral resolution was 4 cm^−1^, and the number of scans was 64 for each spectrum.

Due to their intrinsic orientation, the Raman and IR spectra were recorded by positioning the fibers along one specific direction. Three Raman/IR spectra at least were recorded on different positions of each sample.

The Raman and ATR-IR spectroscopies were applied to the study of hair locks to gain complementary information on the composition of the fibers. In fact, the former is sensitive to the sample bulk (and thus to the cortical region of hair), the latter to the surface skin (and thus to the cuticle region).

### 2.4 Vibrational spectra processing

The average Raman spectra were then processed to obtain information on hair conformational changes and the formation of sulfur oxidation products. The area ratios of selected bands were used to evaluate the relative contents of several compounds and structures after drawing an appropriate baseline by using the software Spectra Manager, version 1.53.03, Jasco Corporation.

Raman area ratios used in the study:

• A_s-s_/A_1450_, A_1040_/A_1450_, A_s-s_/A_1004,_ and A_1040_/A_1004_, where A_s-s_, A_1040_, A_1450,_ A_1004_ were the areas of the bands assignable to disulfide bridges, cysteic acid at about 1040 cm^−1^, CH_2_ bending at 1450 cm^−1^, and phenylalanine at 1004 cm^−1^. Band areas were calculated from the peak to a linear baseline; for A_s-s_, A_1040_, A_1450,_ A_1004_ the baseline was drawn between 482 and 585 cm^−1^, between 1070 and 1020 cm^−1^, between 1500 and 1375 cm^−1^, and between 1013 and 993 cm^−1^, respectively. For more confident results, both 1450 cm^−1^ (bending CH_2_) and 1004 cm^−1^ (phenylalanine) bands were used as internal standards since hair treatments were both supposed to be affected. These ratios were used to estimate the relative contents of disulfide bridges and cysteic acid (as sulfonate salt, R-SO_3_^−^).
• A_935_/A_1450_ ratio, where A_935_ was the area of the band at 935 cm^−1^ assignable to α-helix skeletal C-C stretching [2] (calculated drawing a baseline between 912 and 951 cm^−1^). This ratio allowed us to evaluate the relative α-helix content.

The 1740–1570 cm^−1^ (Amide I) and 580–470 cm^−1^ spectral ranges were analyzed by a curve-fitting procedure to evaluate the content of secondary structures (according to a consolidated method [16,17]) and the conformation of the C_α_-C_β_-S-S-C_β_-C_α_ linkage in cystine disulfide bridges, respectively. The curve fitting analysis requires some preliminary elaborations of the spectra:

• A linear correction in the above-mentioned spectral ranges brought the baseline of the Raman spectra to approximately zero intensity.
• The frequencies of the maxima of the fourth-derivative spectra (obtained with 13-point smoothing) were used as starting positions for the curve-fitting procedure.

The curve-fitting analysis was performed using the OPUS version 6.5 program, using the Levenberg–Marquardt algorithm. The Raman component profiles were described as a linear combination of Lorentzian and Gaussian functions: an FHWM of 8 cm^−1^ with a combination of 10% Lorentzian and 90% was used as the starting parameters. The software guesses the initial intensity of the bands. The content of α-helix, β-sheet, β-turns, and unordered conformations was calculated from the area of the individually assigned bands (at about 1655, 1670, 1685, and 1640 cm^-1^, respectively) [2] and expressed as a fraction of the total area. The obtained areas represent the percentage of secondary conformations, in the generally accepted hypothesis [18] that the Raman cross-section is the same for all the amide I modes.

Similarly, the contents of strained, *gauche-gauche-gauche, gauche-gauche-trans* and *trans-gauche-trans* C_α_-C_β_-S-S-C_β_-C_α_ conformations were determined from the areas of the bands at about 495, 505, 520, and 540 cm^-1^, respectively [2]. The content of each conformation was calculated from the area of the individually assigned bands and expressed as a fraction of the total area of the above-mentioned bands.

The average ATR spectra were then processed to obtain information on hair conformational changes and the formation of sulfur oxidation products. The area and the intensity ratios of selected bands were used to evaluate the relative contents of several compounds and structures after drawing an appropriate baseline by using the software Spectra Manager, version 1.53.03, Jasco Corporation.

IR intensity ratios used in the study:

• The relative content of cysteic acid (as sulfonate salt) was evaluated through the I_1040_/I_Amide I_ and I_1175_/I_Amide I_ ratios, where I_1040,_ I_1175,_ and I_Amide I_ were the absorbances (measured as peak heights) of the cysteic acid bands at about 1040 and 1175 cm^−1^ [11] and Amide I, used as internal standard. Peak heights were calculated from the peak maxima to a linear baseline; for the bands at 1040 and 1175 cm^−1^, baseline was drawn between 1330 and 946 cm^−1^, for Amide I it was drawn between 1724 and 1348 cm^−1^. We preferred to calculate ratios as peak heights rather than peak areas since the bands at 1040 and 1175 cm^−1^ (see in particular the spectrum of brown hair) both belong to a complex envelope of bands and thus the former method appeared more reliable than the latter.

IR area ratio used in the study:

• The Bunte salt/cysteic acid ratio was evaluated through the A_1025_/A_1040_ ratio, where A_1025_ and A_1040_ were the areas of the bands at about 1025 cm^−1^, assignable to the Bunte salt (R-S-SO_3_^−^) [12], and 1040 cm^−1^, assignable to the cysteic acid, respectively. Since these bands can be considered two components of a broader band, areas were determined by a curve fitting procedure after subtracting a baseline in the 1090–960 cm^−1^ interval and using the frequencies of the maxima of the fourth-derivative spectra (obtained with 13-point smoothing) as starting positions for the curve-fitting procedure. The IR component profiles were described as pure Gaussian functions.

### 2.5 Statistical analysis

Statistical analysis on Raman and IR data was performed with *R* statistical software (version 3.5.3; GNU GPL license). The data have a non-Gaussian distribution, so a non-parametric Kruskal–Wallis test was used for the statistical significance (set at *P* < 0.05), and a Dunn–Bonferroni post-hoc analysis has been performed for any dependent variable for which the Kruskal–Wallis test was significant. The Kruskal–Wallis test does not compare means but is based on ranks and was used to verify if the rank means are different. Nevertheless, we reported the data as average values with their associated standard deviation (SD) for better readability.

## CRediT Author Statement

**Michele Di Foggia:** Formal analysis, Writing – original draft, Data curation, Investigation; **Carla Boga:** Conceptualization, Supervision; **Gabriele Micheletti** and **Benedetta Nocentini:** Investigation, Methodology; **Paola Taddei:** Formal analysis, Writing – original draft, Data curation, Investigation, Methodology, Writing - review & editing.

## Declaration of Competing Interest

The authors declare that they have no known competing financial interests or personal relationships which have or could be perceived to have influenced the work reported in this article.

## Appendix Supplementary materials

Image, application 1
